# Supplementary figures and images for: Three-year hospital-wide pain management system implementation at a tertiary medical center: Pain prevalence analysis
Source: PLoS One. 2023 Apr 13;18(4):e0283520. doi: 10.1371/journal.pone.0283520 (PMC10101381; doi:10.1371/journal.pone.0283520)

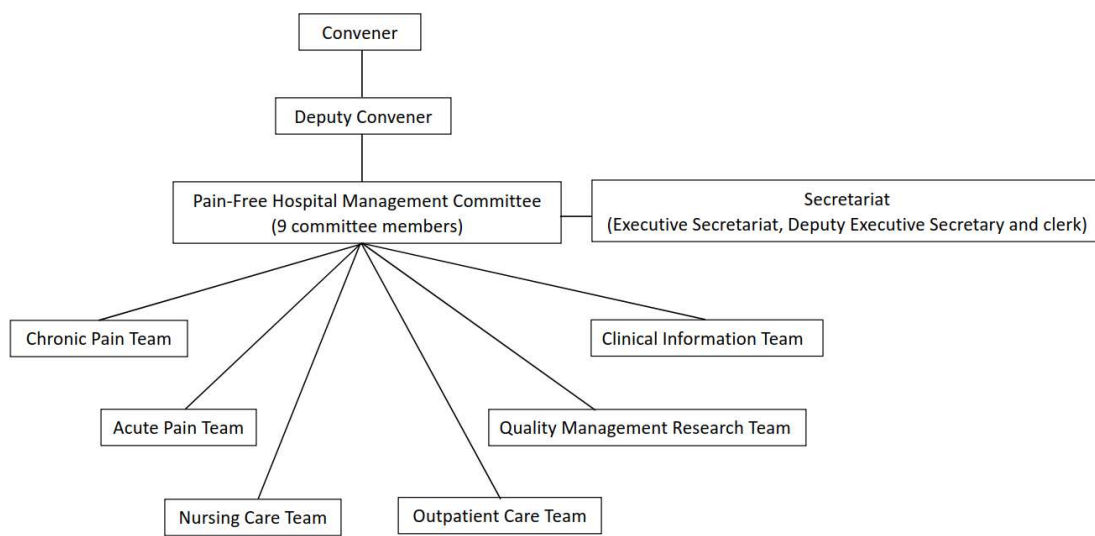

**S1 Fig. The organizational structure of the Pain-Free Hospital Committee**

Supplement: S1 Fig — (PDF) [file pone.0283520.s001.pdf]
