# Supplementary material for: Three-year hospital-wide pain management system implementation at a tertiary medical center: Pain prevalence analysis
Source: PLoS One. 2023 Apr 13;18(4):e0283520. doi: 10.1371/journal.pone.0283520 (PMC10101381; doi:10.1371/journal.pone.0283520)
Supplement: S2 Fig — We use the 3324 dashboard system to track patients’ pain status and current prescriptions in real time, and pain experts could leave recommendations in the system after evaluation. This helped us improve the efficiency of pain management. (PDF) [file pone.0283520.s002.pdf]

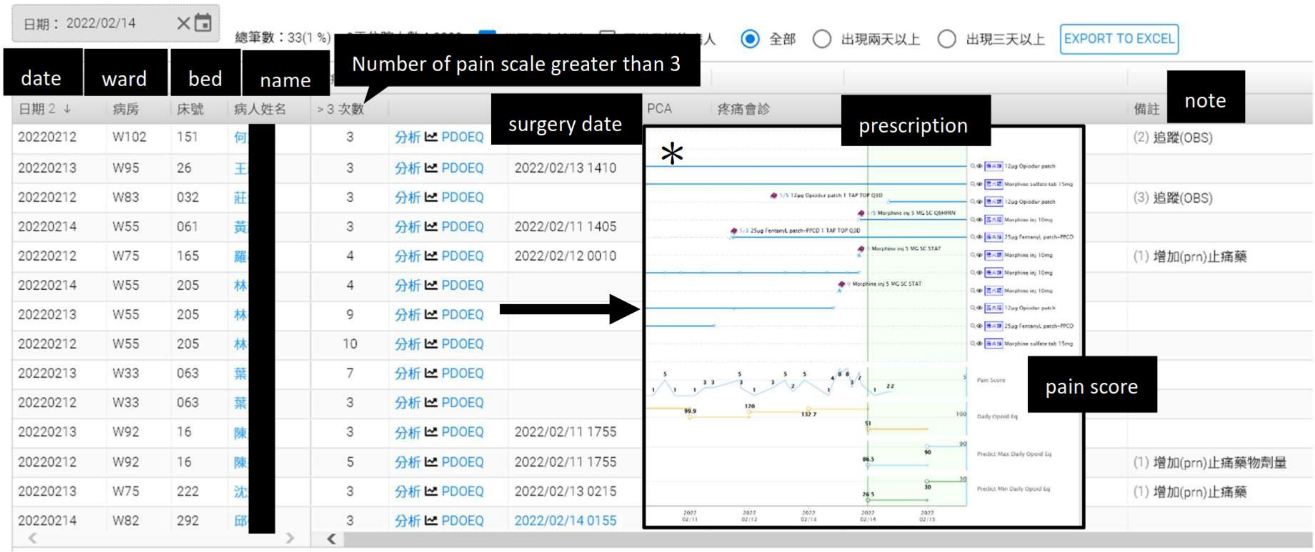

S2 Fig. 3324 Dashboard system. We use the 3324 dashboard system to track patients' pain status and current prescriptions in real time, and pain experts could leave recommendations in the system after evaluation. This helped us improve the efficiency of pain management.
