# Supplementary material for: Three-year hospital-wide pain management system implementation at a tertiary medical center: Pain prevalence analysis
Source: PLoS One. 2023 Apr 13;18(4):e0283520. doi: 10.1371/journal.pone.0283520 (PMC10101381; doi:10.1371/journal.pone.0283520)
Supplement: S1 Table — Pain-free hospital group meetings are held regularly every two weeks to set up cross-team member participation, establish communication channels, and invite team members to discuss care implementation strategies. Monthly case discussions and ward team meetings were conducted for outpatient cases with special or refractory pain. The management committee discussed the business promotion, the work report of each group, the development of the annual policy, and the progress review on a quarterly basis, and reported the implementation progress to the hospital every six months. (PDF) [file pone.0283520.s003.pdf]

Conference name	Frequency of meeting	Discussion items
**Pain-free Hospital Group Meeting**	every two weeks	1. tracking matters in the previous period<br>2. content and progress of the annual plan<br>3. monitoring indicators include business volume and pain-related process and outcome<br>4. presenting the results of special cases of pain during the month
**Pain Special Case Seminar**	monthly	Pain physicians discuss cases of special or refractory pain in outpatient clinics.
**Pain care discussion in ward team meeting**	monthly	Each ward holds monthly ward team meetings, and ward doctors and nurses will discuss special cases of pain and pain management in the ward this month.
**Painless Hospital Management Conference**	quarterly	1. business promotion from the secretariat<br>2. each team work report<br>3. annual policy formulation and progress review
**Hospital Conference**	every six months	Report to the dean on the implementation

S1 Table. Conference for review of pain management system. Pain-free hospital group meetings are held regularly every two weeks to set up cross-team member participation, establish communication channels, and invite team members to discuss care implementation strategies. Monthly case discussions and ward team meetings were conducted for outpatient cases with special or refractory pain. The management committee discussed the business promotion, the work report of each group, the development of the annual policy, and the progress review on a quarterly basis, and reported the implementation progress to the hospital every six months.
