# Supplementary material for: Three-year hospital-wide pain management system implementation at a tertiary medical center: Pain prevalence analysis
Source: PLoS One. 2023 Apr 13;18(4):e0283520. doi: 10.1371/journal.pone.0283520 (PMC10101381; doi:10.1371/journal.pone.0283520)
Supplement: S1 Appendix — (PDF) [file pone.0283520.s004.pdf]

Taichung Veterans General Hospital [Inpatient] Patient Pain Care Experience Survey

English version.

In order to improve the quality of medical services and establish a high-quality medical environment, the Nursing Department, the Anesthesia Department and the Quality Management Center of our hospital jointly invite you to fill out this questionnaire. The purpose is to explore your experience in pain care, and the results of the questionnaire will be analyzed as a reference for the improvement of "Towards a Pain-Free Hospital" program. Your valuable comments will be our motivation for progress. Thank you for your support and assistance.

Taichung Veterans General Hospital

I. Personal information:

1. Gender: ☐ male ☐ female
2. Age:_________years old
3. Weight:__________kg
4. Education level: ☐ elementary school ☐ junior high school ☐ senior high school ☐ college or above ☐ illiterate
5. Marital status: ☐ unmarried ☐ married ☐ divorced, separated ☐ widowed
6. Admission department: ________________________________
7. Have you had surgery during this hospital stay? ☐ Yes ☐ No
8. What is your anesthesia for your surgery?

☐ general anesthesia ☐ regional anesthesia ☐ local anesthesia

9. Use of PCA (patient-controlled analgesia): ☐ Yes ☐ No
10. PCA Route of Administration:

☐ Intravenous Administration ☐ Epidural Administration

II. Pain experience

1. During this hospitalization, do you have pain?

☐ Yes (please continue to answer the pain score) ☐ None

What is your pain scale?

0-----1-----2-----3-----4-----5-----6-----7-----8-----9-----10

2-1. Do you have pain in the past 24 hours:

☐ Yes (please fill in the following) ☐ No

2-2. Duration of pain in the past 24 hours

☐ less than 1 hour☐ 1 to 4 hours☐ 4 to 8 hours☐ 8 to 24 hours

3. Please circle up: How much pain affects my following activities?

(0 point stands for completely non-disruptive, 10 points stand for very disturbing):

(1) On the bed: such as turning over, sitting up and moving positions

0-----1-----2-----3-----4-----5-----6-----7-----8-----9-----10

(2) Walking

0-----1-----2-----3-----4-----5-----6-----7-----8-----9-----10

(3) During breaks

0-----1-----2-----3-----4-----5-----6-----7-----8-----9-----10

(4) Sleeping

0-----1-----2-----3-----4-----5-----6-----7-----8-----9-----10

4. Please circle up: How much pain affects my mood?

(0 point stands for no impact, 10 points stand for drastic impact)

(1) Anxiety/depression

0-----1-----2-----3-----4-----5-----6-----7-----8-----9-----10

(2) Fear/helplessness

0-----1-----2-----3-----4-----5-----6-----7-----8-----9-----10

(3) Angry

0-----1-----2-----3-----4-----5-----6-----7-----8-----9-----10

5. What is the medical treatment that makes me feel pain during hospitalization?

☐ changing dressing of wound ☐ nasogastric tube ☐ Foley catheter☐ chest tube ☐ injections ☐ changing position and chest percussion ☐ rehabilitation ☐ place a central venous catheter ☐ other___________

III. Perception of pain care from nurses

1. Did the nurse assess my pain?☐ Yes ☐ No

2. How did the nurse ask about the intensity of my pain?

☐ Numerical Rating scale: 0 means no pain at all, 10 means the worst pain.

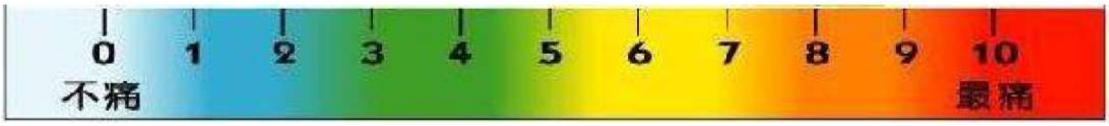

☐ Verbal Rating Scale (VRS): Express the intensity of pain with adjectives such as no pain, mild pain, moderate pain, severe pain, etc.

3. When the nurse evaluates the pain, does he/she ask me about the pain scale when I rest or move?☐ Yes ☐ No

4. When does the nurse provide patient instructions about pain?

☐ no instruction provided ☐ admission ☐ discharge ☐ in pain ☐ getting out of bed ☐ before surgery ☐ after surgery ☐ other _______________

5. When I reported a pain problem, how soon would the nurse assess my pain?

☐ I did not report pain

☐ 15 minutes☐ 15-30 minutes☐ 30-60 minutes ☐ more than 60 minutes

6. After the nursing staff assessed my pain problem, how long would it take to wait for medication?

☐ I did not use painkillers

☐ within 15 minutes ☐ 15 to 60 minutes ☐ more than 60 minutes

7. When the nurse gives painkillers, there is a description of the purpose and instruction?

☐ always ☐ sometimes ☐ never

8. Does the dosage of painkillers meet my needs?

☐ Yes ☐ too much for me ☐ too less for me ☐ I don't know

9. After taking pain medications, my nurse asked me if I had side effects.

☐ always ☐ sometimes ☐ never

10. After taking pain medication, do I have the following side effects?

(If not, circle "0." If so, circle a number that stands for severity (the higher the number, the more serious the problem.))

Nausea 0----- 1----- 2-----3----- 4----- 5----- 6----- 7----- 8----- 9----- 10

Drowsiness 0-----1-----2-----3----- 4----- 5----- 6----- 7-----8-----9-----10

Itchy skin 0-----1----- 2-----3----- 4----- 5-----6----- 7----- 8-----9----- 10

Dizziness 0----- 1----- 2----- 3----- 4----- 5----- 6----- 7----- 8-----9-----10

IV. Perception of pain care from physicians

1. Did the physician assess my pain? ☐ Yes☐ No

2. How did the physician ask about the intensity of my pain?

☐ Numerical Rating scale: 0 means no pain at all, 10 means the worst pain.

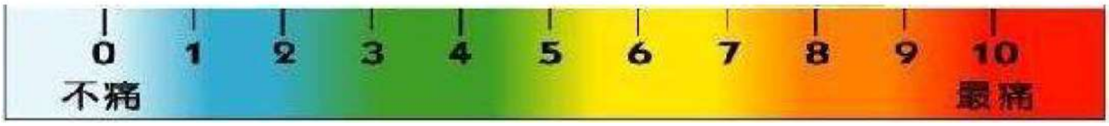

☐ Verbal Rating Scale (VRS): Express the intensity of pain with adjectives such as no pain, mild pain, moderate pain, severe pain, etc.

3. When the physician evaluates the pain, does he/she ask me about the pain scale when I rest or move? ☐ Yes ☐ No

4. When the physician prescribes painkillers, there is a description of the purpose and instruction?

☐ always ☐ sometimes ☐ never

5. After taking pain medications, my physician asked me if I had side effects?

☐ always ☐ sometimes ☐ never

V. Overall satisfaction

1. How do you feel about the pain management provided by our physicians?

☐ very satisfactory ☐ satisfactory ☐ average ☐ unsatisfactory ☐ very unsatisfactory

2. How do you feel about the pain management provided by our nursing staff?

☐ very satisfactory ☐ satisfactory ☐ average ☐ unsatisfactory ☐ very unsatisfactory

3. For overall pain management in our hospital, I think it is

☐ very satisfactory ☐ satisfactory ☐ average ☐ unsatisfactory ☐ very unsatisfactory
