# Supplementary material for: Three-year hospital-wide pain management system implementation at a tertiary medical center: Pain prevalence analysis
Source: PLoS One. 2023 Apr 13;18(4):e0283520. doi: 10.1371/journal.pone.0283520 (PMC10101381; doi:10.1371/journal.pone.0283520)
Supplement: Attachment — Submitted filename: PONE-D-22-25681_CAS.pdf [file pone.0283520.s006.pdf]

PLOS ONE

Three-year hospital-wide pain management system implementation at a tertiary medical center: pain prevalence analysis

--Manuscript Draft--

**Manuscript Number:**	PONE-D-22-25681
**Article Type:**	Research Article
**Full Title:**	Three-year hospital-wide pain management system implementation at a tertiary medical center: pain prevalence analysis
**Short Title:**	Pain management system and pain prevalence analysis
**Corresponding Author:**	Ching-Hui Shen<br>Taichung Veterans General Hospital<br>Taichung, TAIWAN
**Keywords:**	Towards a Pain-Free Hospital; information dashboard system; audits and feedback; pain prevalence
**Abstract:**	We developed a pain management system over a 3-year period. In this project, “Towards a pain-free hospital”, we combined evidence-based medicine and medical expertise to develop a series of policies. To gain an understanding of the changes in the prevalence of pain in inpatients under the care of the pain management system. The subjects of the survey are inpatients over 20 years old, and who had been hospitalized in the general ward for at least 3 days. The patients would be excluded if they were unable to respond to the questions. We randomly select eligible patients in the general ward. Our trained interviewers visited inpatients to complete the questionnaires designed by our pain care specialists. A total of 3,094 inpatients completed the survey from 2018 to 2020. The prevalence rates of pain experienced by the patients in the 3-year period were 69.5% (2018) (reference), 63.3% (2019) (OR:0.768, p<0.01), and 60.1% (2020) (OR:0.662, p <0.001). The prevalence rates of pain in patients undergoing surgery during the 3-year period were 81.4% (2018), 74.3% (2019), and 68.8% (2020), respectively. As for care-related causes of pain, injection, change in position/chest percussion, and rehabilitation showed a decreasing trend over the 3-year period of study. Our pain management system provided immediate professional pain management, and achieved a good result in the management of acute moderate to severe pain, especially perioperative pain. Studies on pain prevalence and Pain-Free Hospitals are scarce in Asia. With the aid of the policies based on evidence-based medicine and the dashboard information system, from 2018 to 2020, the prevalence of pain has decreased year by year.
**Order of Authors:**	Ming-Chuan Chen
	Te-Feng Yeh
	Chih-Cheng Wu
	Yan-Ru Wang
	Chieh-Liang Wu
	Ching-Hui Shen
**Opposed Reviewers:**	
**Additional Information:**	
**Question**	**Response**
**Financial Disclosure**<br><br>Enter a financial disclosure statement that describes the sources of funding for the work included in this submission. Review the submission guidelines for detailed	The author(s) received no specific funding for this work.

Powered by Editorial Manager® and ProduXion Manager® from Aries Systems Corporation

requirements. View published research articles from *PLOS ONE* for specific examples.<br><br>This statement is required for submission and **will appear in the published article** if the submission is accepted. Please make sure it is accurate.<br><br>**Unfunded studies**<br>Enter: *The author(s) received no specific funding for this work.*<br><br>**Funded studies**<br>Enter a statement with the following details:<br>• Initials of the authors who received each award<br>• Grant numbers awarded to each author<br>• The full name of each funder<br>• URL of each funder website<br>• Did the sponsors or funders play any role in the study design, data collection and analysis, decision to publish, or preparation of the manuscript?<br>• **NO** - Include this sentence at the end of your statement: *The funders had no role in study design, data collection and analysis, decision to publish, or preparation of the manuscript.*<br>• **YES** - Specify the role(s) played.<br><br>* typeset	
**Competing Interests**<br><br>Use the instructions below to enter a competing interest statement for this submission. On behalf of all authors, disclose any competing interests that could be perceived to bias this work—acknowledging all financial support and any other relevant financial or non-financial competing interests.<br><br>This statement is required for submission and **will appear in the published article** if the submission is accepted. Please make sure it is accurate and that any funding sources listed in your Funding Information later in the submission form are also declared in your Financial Disclosure	The authors have declared that no competing interests exist.

statement.<br><br>View published research articles from *PLOS ONE* for specific examples.<br><br>**NO authors have competing interests**<br><br>Enter: *The authors have declared that no competing interests exist.*<br><br>**Authors with competing interests**<br><br>Enter competing interest details beginning with this statement:<br><br>*I have read the journal's policy and the authors of this manuscript have the following competing interests: [insert competing interests here]*<br><br>* typeset	
**Ethics Statement**<br><br>Enter an ethics statement for this submission. This statement is required if the study involved:<br><br>• Human participants<br>• Human specimens or tissue<br>• Vertebrate animals or cephalopods<br>• Vertebrate embryos or tissues<br>• Field research<br><br>Write "N/A" if the submission does not require an ethics statement.<br><br>General guidance is provided below. Consult the submission guidelines for detailed instructions. **Make sure that all information entered here is included in the Methods section of the manuscript.**	This research was approved by the Institutional Review Board I & II of Taichung Veterans General Hospital (Certificate Number: CE18236B). After the patients signed the informed consent document, we started our questionnaire.

Powered by Editorial Manager® and ProduXion Manager® from Aries Systems Corporation

**Format for specific study types**<br><br>**Human Subject Research (involving human participants and/or tissue)**<br>• Give the name of the institutional review board or ethics committee that approved the study<br>• Include the approval number and/or a statement indicating approval of this research<br>• Indicate the form of consent obtained (written/oral) or the reason that consent was not obtained (e.g. the data were analyzed anonymously)<br><br>**Animal Research (involving vertebrate animals, embryos or tissues)**<br>• Provide the name of the Institutional Animal Care and Use Committee (IACUC) or other relevant ethics board that reviewed the study protocol, and indicate whether they approved this research or granted a formal waiver of ethical approval<br>• Include an approval number if one was obtained<br>• If the study involved *non-human primates*, add *additional details* about animal welfare and steps taken to ameliorate suffering<br>• If anesthesia, euthanasia, or any kind of animal sacrifice is part of the study, include briefly which substances and/or methods were applied<br><br>**Field Research**<br><br>Include the following details if this study involves the collection of plant, animal, or other materials from a natural setting:<br>• Field permit number<br>• Name of the institution or relevant body that granted permission	
**Data Availability**<br><br>Authors are required to make all data underlying the findings described fully available, without restriction, and from the time of publication. PLOS allows rare exceptions to address legal and ethical concerns. See the PLOS Data Policy and FAQ for detailed information.	Yes - all data are fully available without restriction

A Data Availability Statement describing where the data can be found is required at submission. Your answers to this question constitute the Data Availability Statement and **will be published in the article**, if accepted.<br><br>**Important:** Stating 'data available on request from the author' is not sufficient. If your data are only available upon request, select 'No' for the first question and explain your exceptional situation in the text box.<br><br>Do the authors confirm that all data underlying the findings described in their manuscript are fully available without restriction?	
**Describe where the data may be found in full sentences. If you are copying our sample text, replace any instances of XXX with the appropriate details.**<br><br>• If the data are **held or will be held in a public repository**, include URLs, accession numbers or DOIs. If this information will only be available after acceptance, indicate this by ticking the box below. For example: *All XXX files are available from the XXX database (accession number(s) XXX, XXX.*).<br>• If the data are all contained **within the manuscript and/or Supporting Information files**, enter the following: *All relevant data are within the manuscript and its Supporting Information files.*<br>• If neither of these applies but you are able to provide **details of access elsewhere**, with or without limitations, please do so. For example:<br><br>*Data cannot be shared publicly because of [XXX]. Data are available from the XXX Institutional Data Access / Ethics Committee (contact via XXX) for researchers who meet the criteria for access to confidential data.*<br><br>*The data underlying the results presented in the study are available from (include the name of the third party*	All relevant data are within the manuscript and its Supporting Information files.

and contact information or URL).<br>• This text is appropriate if the data are owned by a third party and authors do not have permission to share the data.<br><br>* typeset	
Additional data availability information:	

Dear Editors,

We would like to submit the enclosed manuscript entitled “Three-year hospital-wide pain management system implementation at a tertiary medical center: pain prevalence analysis” which we would like to submit for publication as a Research Article in PLoS One.

We have implemented the "Towards a Pain-Free Hospital" program since 2018 and developed a series of measures, including the development of standard procedures for inpatient pain management, the implementation of hospital-wide pain medicine education and training, the establishment of a dashboard system to track pain status, and regular audits and feedback. This study investigated the pain status of inpatients by means of questionnaires and aimed to gain an understanding of the changes in the prevalence of pain in inpatients under the care of the pain management system during the 3-year period of implementation and to analyze the associated factors.

We found a gradual decrease in the prevalence of pain over the 3-year period. In the subgroup analysis, the reduction was even more significant in the groups with acute pain and moderate to severe pain. Furthermore, pain caused by injection, change in position/chest percussion, and rehabilitation showed a decreasing trend over the 3-year period of study.

Pain is one of the most common clinical problems. The promotion of pain management quality is beneficial to the patient's physical and mental status and satisfaction with the medical treatment process. In the program, by virtue of our (1) a multidisciplinary team, (2) an evidence-based education program, (3) a data-driven Information Dashboard and (4) regular audits and feedback, there is a significant downward trend in the prevalence of pain in 3 years. This study is of interest to researchers, clinicians and hospital administrators working on pain management. We hope to promote a systematic and evidence-based approach to improve the quality of pain management in hospitals worldwide. Therefore, we feel this manuscript is particularly suitable for PLoS One and of great interest to its readers.

We would like to suggest the following Academic Editors for our manuscript

1. Hsin-Yen Yen, Taipei Medical University, TAIWAN
2. Xiaohui Wang, University of the Chinese Academy of Sciences, CHINA
3. Stefano Triberti, University of Milan, ITALY
4. Paavani Atluri, Bay Area Hospital, North Bend Medical Center, UNITED STATES
5. Pradeep Nair, Central University of Himachal Pradesh, INDIA

If you require any additional information regarding our manuscript, please contact us directly via the resources below. Thank you for your time and consideration.

Sincerely,

Dr. Ming-Chuan Chen
chen510156@gmail.com

Manuscript Click here to access/download;Manuscript;Manuscript.docx

Three-year hospital-wide pain management system implementation at a tertiary medical center: pain prevalence analysis

Ming-Chuan Chen[1], Te-Feng Yeh[2], Chih-Cheng Wu[1,3,4], Yan-Ru Wang[2], Chieh-Liang Wu[5], Ching-Hui Shen[1,6,7]*

[1]Department of Anesthesiology, Taichung Veterans General Hospital, Taichung, Taiwan

[2]Department of Healthcare Administration, Central Taiwan University of Science and Technology, Taichung, Taiwan

[3]Department of Financial Engineering, Providence University, Taichung, Taiwan

[4]Department of Data Science and Big Data Analytics, Providence University, Taichung, Taiwan

[5]Center of Smart Healthcare, Taichung Veterans General Hospital, Taichung, Taiwan

[6]School of Medicine, National Yang Ming Chiao Tung University, Taipei, Taiwan

[7]Department of Post-Baccalaureate Medicine, College of Medicine, National Chung Hsing University, Taichung, Taiwan

* Corresponding author:

E-mail: shench07@gmail.com (CHS)

Abstract

We developed a pain management system over a 3-year period. In this project, “Towards a pain-free hospital”, we combined evidence-based medicine and medical expertise to develop a series of policies. To gain an understanding of the changes in the prevalence of pain in inpatients under the care of the pain management system. The subjects of the survey are inpatients over 20 years old, and who had been hospitalized in the general ward for at least 3 days. The patients would be excluded if they were unable to respond to the questions. We randomly select eligible patients in the general ward. Our trained interviewers visited inpatients to complete the questionnaires designed by our pain care specialists. A total of 3,094 inpatients completed the survey from 2018 to 2020. The prevalence rates of pain experienced by the patients in the 3-year period were 69.5% (2018) (reference), 63.3% (2019) (OR:0.768, $p<0.01$), and 60.1% (2020) (OR:0.662, $p <0.001$). The prevalence rates of pain in patients undergoing surgery during the 3-year period were 81.4% (2018), 74.3% (2019), and 68.8% (2020), respectively. As for care-related causes of pain, injection, change in position/chest percussion, and rehabilitation showed a decreasing trend over the 3-year period of study. Our pain management system provided immediate professional pain management, and achieved a good result in the management of acute moderate to severe pain, especially perioperative pain. Studies on pain prevalence and Pain-Free Hospitals are scarce in Asia. With the aid of the policies based on evidence-based medicine and the dashboard information system, from 2018 to 2020, the prevalence of pain has decreased year by year.

Introduction

Pain is one of the most common complaints of inpatients in the hospital. The prevalence of pain in hospitals in various countries ranges from 52 to 100%.[1-4] We conducted a study of pain prevalence

among inpatients in 2018,[5] and nearly 70% of hospitalized patients experienced pain. Therefore, pain management is an important issue in all medical institutions. Pain-related stress may cause instability in vital signs such as blood pressure and heart rate. It can also worsen a patient's quality of life, and has a deleterious effect on recovery from illness or surgery.[6] Poor pain management leads to longer hospital stays and increased readmissions, resulting in a waste of medical resources.[7] The hospital should provide comprehensive pain management to improve the physical and mental condition of most patients suffering from pain.

"Towards a Pain-Free Hospital" is a campaign that has been implemented in some hospitals in North America and Europe, and its efficacy has also been evaluated. Twenty hospitals in Italy participated in this campaign in 2000.[8] In Germany the campaign was launched in 2003 to improve the effectiveness of pain management. It was found that 55% of German surgical patients and 57% of non-surgical patients were not satisfied with their pain management. The patients believed that effective pain management remained insufficient.[9] The evidence shows pain management does not meet the expectations of hospitalized patients. In addition to developing and updating clinical care guidelines for pain management,[10, 11] it is necessary to develop an effective strategy to overcome the wide gap between clinical care guidelines and real-life care processes.

Evidence-based practices can be achieved with continuous quality management. Auditing and feedback generally lead to small but potentially important improvements in professional practice.[12] Introducing clinical and quality dashboards can have a positive effect on care outcomes and processes of care.[13] Needham DM proposed a model for undertaking knowledge translation: step 1, summarize the evidence; step 2, identify local barriers to implementation; step 3, measure performance; and step 4, ensure that patients have received the intervention.[14] In terms of hospitalized pain management, it is necessary to translate the knowledge of pain management step by step into real-life care with the Plan-Do-Check-Act (PDCA) procedure and conducting effective

auditing and feedback using the indicators shown on the dashboard.

To reach the goal of “a pain-free hospital”, we launched a hospital-wide pain management project in 2018. The project adopted an evidence-based approach with a data-driven information system to control pain. We surveyed patients’ pain prevalence from 2018 to 2020. A detailed description of the project and the methods that were used to successfully improve pain management among the hospitalized patients is provided below.

Methods

Study setting

Taichung Veterans General Hospital is a medical center located in central Taiwan. There are 19 general wards in our hospital, with about 50,000 hospitalizations per year. We implemented a hospital-wide pain management campaign in 2018, and surveyed the pain prevalence of inpatients from 2018 to 2020. This research was approved by the Institutional Review Board I & II of Taichung Veterans General Hospital (Certificate Number: CE18236B).

The project of hospital-wide pain management

The initial preparation for the pain management project in our hospital commenced in 2017. First, we organized pain management committees and recruited specialists in pain management, which included a nursing care team, an education team, computer technicians, a quality improvement team, and the leadership (S1 Fig). Second, we reviewed the recent literature to update our guidelines for pain management.[15-17] Third, we developed teaching materials based on our guidelines and launched the hospital-wide education program in May, 2018. We trained the seed instructors to

provide lectures for each division and each ward. The training courses and lectures in each unit were repeated every year. We also provided feedback on the achievement of each unit during the lecture. Fourth, we designed our daily pain indicator "3324" which is detailed in the following section. Our computer and medical informatics engineers designed the pain management system and dashboard (S2 Fig), which integrate the nurses' measurements of the patients' pain levels, pain medications, and the alarm of 3324. Fifth, using the PDCA concept, the indicators of the process and outcome in the pain management were reviewed by the team of the participating wards, divisions, committees, and the hospital at the indicated periods (S1 Table).

The response cycle of the real-time pain indicator of "3324"

We conducted a review of the literature pertaining to "3-3-3", which is for chronic pain management, and based on the findings, we designed an approach termed "3324". This number means that the scale of Visual Analogue Scale (VAS) was > 3 and occurred ≥ 3 times within the last 24 hours during hospitalization. The signal of 3324 is shown on the Dashboard Pain Management System, and the nurses and physicians can see it on the working panel of the electronic medical record system. The medical staff then take the necessary actions to control the patients' pain following the process (Fig 1). If the patient still has refractory pain, the case manager of pain (a nurse) can visit the patient and consult with a pain specialist (anesthesiology) to adjust the analgesic prescription or consider regional analgesic therapies, such as peripheral nerve block.

Fig. 1. Standard Process for Inpatient Pain Management. Based on clinical care guidelines and evidence-based medicine, we developed the "Standard Process for Inpatient Pain Management", and set up quality indicators from pain management process for auditing and feedback.

*PCA: patient-controlled analgesia

Pain prevalence survey

We surveyed the pain prevalence of inpatients from 2018 to 2020, according to the method described in our previous research.[5] We used a questionnaire that is divided into 4 main parts, including (1) personal information, (2) pain experience during hospitalization, (3) perception of pain care from physicians and nurses, and (4) overall satisfaction (S1 Appendix). The patients (1) were at least twenty years old, (2) had a hospital stay of at least thr[illegible]days, (3) had clear consciousness, and (4) provided consent to be included in this study. Patients were excluded if they were not able to express themselves e.g., had dementia, brain injury, or critical illness. The interviewers conducted a face-to-face questionnaire interview in the ward. We recruited inpatients using quota sampling for each general ward. Enrollment will be stopped when each general ward reaches the quota sampling number of the day. To implement standardized interviews, we train interviewers before conducting the questionnaires. On ward visits, the nurses listed eligible patients, and we randomly sampled and interviewed patients. After the patients signed the informed consent document, we started our questionnaire.

Statistics

All of the patient information was anonymized before analysis. We used numeric rating scale to classify pain levels into mild (1-3), moderate (4-6), and severe (7-10). We determined the factors related to pain by the chi-squa[illegible]est and multiple logistic regression. An alpha level of $p < 0.05$ was considered statistically significant. All analyses were performed using SPSS Windows version 21.0.

Results

3-year demographic data:

To evaluate the outcome of the hospital-wide pain management project, we surveyed the patients' pain prevalence from 2018 to 2020 according to a previously published method.[5] In total, 1034, 1035, and 1025 patients were recruited by quota sampling from wards in 2018, 2019, and 2020, respectively. In 2020, we followed the same collection pattern as the previous year. However, because of COVID-19 outbreak, part of the ward was converted into a negative pressure isolation room. The total number of general beds was reduced by 10, so 1,025 samples were collected. Therefore, a total of 3,094 samples were collected in the three years. The demographic data of all of the patients are shown in Table 1. Among all patients, males comprised 54.3% and the elderly (>60 years) accounted for 52.7%. Approximately 41.5% and 47.3% of these patients were admitted to the surgical and medical departments, respectively. Notably, more than half (51.1%) of all patients received surgical operations. There was no difference in the demographic data during the three-year survey, except for age, weigh[illegible]nd marital status.

Table1. Demographic data of the patients from 2018 to 2020

Characteristics	ALL	2018	2019	2020	*p*-value
	N=3,094 (%)	N=1,034 (%)	N=1,035 (%)	N=1,025 (%)	
Gender					
Female	1,415 (45.7)	497 (48.1)	458 (44.3)	460 (44.9)	
Male	1,679 (54.3)	537 (51.9)	577 (55.7)	565 (55.1)	
**Age (years)**					**<0.05**
**≤39**	**447 (14.1)**	**168 (16.2)**	**138 (13.3)**	**141 (13.8)**	
**40~49**	**374 (12.1)**	**121 (11.7)**	**119 (11.5)**	**134 (13.1)**	
**50~59**	**643 (20.8)**	**240 (23.2)**	**208 (20.1)**	**195 (19.0)**	
**60~69**	**789 (25.5)**	**257 (24.9)**	**258 (24.9)**	**274 (26.7)**	
**70~79**	**508 (16.4)**	**136 (13.2)**	**194 (18.7)**	**178 (17.4)**	
**≥80**	**333 (10.8)**	**112(10.8)**	**118 (11.4)**	**103 (10.0)**	
**Weight (kg)**					**<0.01**
**≤50**	**435 (14.1)**	**144 (13.9)**	**130 (12.6)**	**161 (15.7)**	
**50~59**	**874 (28.2)**	**277(26.8)**	**291 (28.1)**	**306 (29.9)**	
**60~69**	**966 (31.2)**	**322 (31.1)**	**323 (31.2)**	**321 (31.3)**	

**70~79**	**470 (15.2)**	**186 (18.0)**	**166 (16.0)**	**118 (11.5)**	
**≥80**	**349 (11.3)**	**105 (10.2)**	**125 (12.1)**	**119 (11.6)**	
Education					
Illiterate	215 (6.9)	72 (7.0)	73 (7.1)	70 (6.8)	
Elementary school	673 (21.8)	220 (21.3)	254 (24.5)	199 (19.4)	
Junior high school	460 (14.9)	159 (15.4)	155 (15.0)	146 (14.2)	
Senior high school	848 (27.4)	284 (27.5)	275 (26.6)	289 (28.2)	
Bachelor's degree or higher	898 (29.0)	299 (28.9)	278 (26.9)	321 (31.3)	
**Marriage**					**<0.01**
**Unmarried**	**397 (12.8)**	**142 (13.7)**	**122 (11.8)**	**133 (13.0)**	
**Married**	**2,380 (76.9)**	**765 (74.0)**	**803 (77.6)**	**812 (79.2)**	
**Other**	**317 (10.2)**	**127 (12.3)**	**110 (10.6)**	**80 (7.8)**	
Disease categories					
Surgery	1,284 (41.5)	400 (38.7)	436 (42.1)	448 (43.7)	
Head, Neck, Ophthalmology	155 (5.0)	55 (5.3)	51 (4.9)	49 (4.8)	
Internal Medicine	1,464 (47.3)	509 (49.2)	485 (46.9)	470 (45.9)	
Gynecology	191 (6.2)	70 (6.8)	63 (6.1)	58 (5.7)	
Operation					
No operation	1,514 (48.9)	490 (47.4)	499 (48.2)	525 (51.2)	
With operation	1,580 (51.1)	544 (52.6)	536 (51.8)	500 (48.8)	

N (%): Data are expressed as the case number and its percentage among total cases.

Decreasing the pain prevalence rate after pain management:

The hospital-wide pain management project was first implemented in 2018. As shown in Fig 2, the prevalence rates of pain in 2019 (63.3%) and 2020 (60.1%) were significantly lower than that of 2018 (69.5%) statistically. These prevalence rates between 2019 and 2020 did not reach a significant difference (OR: 0.872, p=0.152). In terms of risk factors that cause pain (Table 2), male gender (OR: 0.821, p<0.05), elderly age (OR: 0.443, p<0.001), and existence of medical diseases (OR: 0.752, p<0.01) were correlated with lower prevalence of pain problem. The rate of pain in patients undergoing surgery was 2.136 times the rate of pain in patients without surgery (p<0.010). We also found that surgery was an important related factor for pain in each of the three consecutive years (OR:2.722, p<0.010) (OR:2.276, p<0.010) (OR:1.640, p<0.010).

Fig. 2. Decrease of pain prevalence after implementing the hospitalized-wide pain management program from 2018 to 2020

Table 2. The changes of risk factor among the patients with pain from 2018 to 2020

Characteristics	ALL	2018	2019	2020
	N=1990 (%)	N=719 (%)	N=655 (%)	N=616 (%)
Gender				
Female	952 (47.8)	353 (49.1)	301 (46.0)	298 (48.4)
Male	**1038 (52.2)**[a]	366 (50.9)	354 (54.0)	**318 (51.6)**[a]
Age (years)				
≤39	337 (16.9)	123 (17.1)	111 (16.9)	103 (16.7)
40~49	**246 (12.4)**[a]	88 (12.2)	**73 (11.1)**[a]	85 (13.8)
50~59	446 (22.4)	174 (24.2)	146 (22.3)	126 (20.5)
60~69	**489 (24.6)**[a]	175 (24.3)	**150 (22.9)**[a]	164 (26.6)
70~79	**295 (14.8)**[a]	95 (13.2)	**107 (16.3)**[a]	**93 (15.1)**[a]
≥80	**177 (8.9)**[a]	64 (8.9)	**68 (10.4)**[a]	**45 (7.3)**[a]
Weight (kg)				
≤50	273 (13.7)	102 (14.2)	76 (11.6)	95 (15.4)
50~59	550 (27.6)	194 (27)	177 (27)	179 (29.1)
60~69	650 (32.7)	223 (31)	**225 (34.4)**[a]	202 (32.8)
70~79	286 (14.4)	120 (16.7)	98 (15.0)	68 (11.0)
≥80	231 (11.6)	80 (11.1)	79 (12.1)	72 (11.7)
Education				
Illiterate	131 (6.6)	46 (6.4)	48 (7.3)	37 (6.0)
Elementary school	391 (19.6)	143 (19.9)	145 (22.1)	103 (16.7)
Junior high school	296 (14.9)	108 (15)	99 (15.1)	89 (14.4)
Senior high school	545 (27.4)	211 (29.3)	164 (25.0)	170 (27.6)
Bachelor’s degree or higher	627 (31.5)	211 (29.3)	199 (30.4)	217 (35.2)
Marriage				
Unmarried	278 (14.0)	100 (13.9)	86 (13.1)	92 (14.9)
Married	1523 (76.5)	543 (75.5)	503 (76.8)	477 (77.4)
Other	189 (9.5)	76 (10.6)	66 (10.1)	47 (7.6)
Disease categories				
Surgery	920 (46.2)	320 (44.5)	302 (46.1)	298 (48.4)
Head, Neck, Ophthalmology	120 (6)	40 (5.6)	**44 (6.7)**[a]	36 (5.8)
Internal Medicine	**803 (40.4)**[a]	**303 (42.1)**[a]	258 (39.4)	242 (39.3)
Gynecology	147 (7.4)	56 (7.8)	51 (7.8)	40 (6.5)
Operation				

No operation	805 (40.5)	276 (38.4)	257 (39.2)	272 (44.2)
With operation	**1185 (59.5)**[a]	**443 (61.6)**[a]	**398 (60.8)**[a]	**344 (55.8)**[a]

N (%): Data are expressed as the case number and its percentage among total cases.

[a]Odds ratio of multiple logistic regression for pain or severe pain significance ($p < 0.05$).

Pain management changed the patients' duration and severity of pain:

Patients with pain during hospitalization (N=719 in 2018, N=655 in 2019 and N=616 in 2020) reported the severity (Fig 3a) and duration (Fig 3b) of pain in the past 24 hours on the day of interview. We used a Numerical Rating Scale (NRS) to divide patients with pain into 4 groups: no pain (0), mild pain (1-3), moderate pain (4-6), and severe pain (7-10). Thirty-three percent of the patients in 2019 reported no pain problems, and the rates of moderate (18.9%) and severe (13.3%) pain were also greatly reduced in comparison with rates in 2018. Although the rates of moderate and severe pain slightly increased in 2020, it was still lower than that in 2018. The duration of pain was divided into 4 groups: transient, less than 1 hour, 1-4 hours, and more than 4 hours. The results showed that the rate of those with longer pain duration increased in 2019 and 2020 in comparison with that in 2018 (Fig 3b).

Fig. 3. The changes of pain severity (a) and pain duration (b) among the hospitalized patients with pain from 2018 to 2020

Care-related pain changed with pain management:

For patients with moderate or severe pain, which lasted for more than 4 hours (N=391), the number

of cases and events are presented in Table 3. Injections (45%), wound dressing (31.7%), change of posture and chest percussion (21.0%) were the most common causes of pain. After the education and training of in-hospital medical staff, the rates of pain related to injections ($p<0.001$), change of posture and chest percussion ($p<0.001$), and rehabilitation ($p<0.05$) were lower in 2019 and 2020 compared with 2018.

Table 3. The changes of care-related pain with severity ≥ 4 and duration ≥ 4 h

Characteristics	ALL	2018	2019	2020	*p*-value
	391 (%)	143 (%)	119 (%)	129 (%)	
**Needle Pain**	**176 (45.0)**	**79 (55.2)**	**58 (48.7)**	**39 (30.2)**	**<0.001**
Wound dressing pain	124 (31.7)	43 (30.1)	41 (34.5)	40 (31.0)	
**Change in posture/Chest percussion**	**82 (21.0)**	**46 (32.2)**	**14 (11.8)**	**22 (17.1)**	**<0.001**
Nasogastric tube	17 (4.3)	10 (7.0)	4 (3.4)	3 (2.3)	
Foley Catheter	22 (5.6)	9 (6.3)	7 (5.9)	6 (4.7)	
**Rehabilitation**	**22 (5.6)**	**10 (7.0)**	**10 (8.4)**	**2 (1.6)**	**<0.05**
Central venous catheter	4 (1.0)	2 (1.4)	0 (0.0)	2 (1.6)	
Chest tube	12 (3.1)	3 (2.1)	4 (3.4)	5 (3.9)	
Drainage tube	1 (0.3)	0 (0.0)	1 (0.8)	0 (0.0)	

Discussion

The pain management project presented herein adopted an evidence-based approach with a data-driven information system to control patients' pain. We designed a novel indicator termed "3324" on the dashboard, which was a real-time PDCA response cycle for the control of pain. After implementing the hospital-wide pain management project, we noted that the prevalence rate of pain decreased from 69.5% to 60.1% over the 3-year period of the study. Though the percentage of patients with long duration of pain was still high, the prevalence rates of pain and the rate of severe pain decreased. As in previous studies, our results indicate that pain remains a major problem in hospitalized patients.[18-22] The results indicate that this project was effective in moving "Towards

a pain free-hospital".

How did this project improve hospital-wide pain management? A few factors contributed to the success of the project. First, we organized a multidisciplinary team and ran the regular activities of the team on wards. Leadership seemed to be pivotal to the success of the project. Second, we conducted a comprehensive education program in all wards and divisions. The educational content was evidence-based and updated every year, taking the shortcomings noted in the previous year into account. For example, we emphasized the importance of care-related pain (rehabilitation and change of posture). We found the rates of rehabilitation-related pain and change of posture-related pain decreased year by year. Third, we applied the concept of continuous quality management using a real-time "3324" PDCA response cycle (Fig 1). Fourth, our pain management protocol was fully supported by the Data-driven Information Dashboard. The system closely integrated the roles of physicians, nurses, head nurses, and case managers.

The project did not change the risk factors of pain among the hospitalized patients, including gender, age, and surgical intervention. The prevalence of pain in females was higher than that in males in our study, which is consistent with the results of many previous studies.[1, 19, 23-34] Falk et al. explained that this may be explained, at least in part, by aspects of traditional culture and gender traits. Males are expected to be brave and may therefore tend to tolerate pain. In contrast, women may be deemed more likely to express emotion and be less able to endure pain compared with men.[34]

In our study, the younger the age, the higher was the prevalence of pain. This finding is in line with previously reported results.[1, 20, 23, 25-27, 35] A study by Gianni et al. investigated elderly patients in eight Italian hospitals and also found that compared with young people, elderly patients did not report their pain problems because they believed that medical staff were busy with clinical work. If they reported pain, they feared that the medical staff would be unhappy with them.[18] The

elderly have limited ability to express pain, so the proportion of actual pain remains high. Therefore, the pain problem of the elderly population cannot be underestimated and ignored.

The pain rate of patients in the department of surgery was significantly higher than that of patients in the department of internal medicine. This is consistent with the findings of Gregory et al.[3] A study by Bourdillon et al. showed that in surgical patients, surgery was a key factor affecting pain.[31] In a survey conducted by Ambrogi et al. in a French university hospital, among 907 patients undergoing surgery, 330 (37%) experienced severe pain. The reason for this was presumed to be the decline of the anesthetic effect in surgical patients and the pain of wound treatment.[21] Other studies on the pain of inpatients showed similar results.[19, 23, 24, 26, 31] These results indicate that surgery is a powerful pain-causing factor. After three years of follow-up, we found that the pain rate of surgical patients decreased year by year. The changes in the pain rate of patients in the department of internal medicine indicate that our pain care measures had a further effect on perioperative pain.

The analysis of pain severity revealed that the proportion of moderate to severe pain decreased, and the proportion of mild pain increased relatively. The duration of pain findings indicate that the proportion of transient pain decreased, and the proportion of pain duration greater than 4 hours relatively increased. Our pain management system was particularly effective for perioperative pain. Postoperative wound pain was acute, and the degree was mostly moderate or severe. With immediate evaluation and treatment, pain can be relieved in a short time. However, the relative proportion of patients with chronic pain and internal medicine has increased. Most of these patients have pain before admission, and some are already receiving routine pain control. The pain of these patients is mostly mild (NRS: 1~3) and persistent. The 3324 system does not warn about this. This means that there is still room for improvement in the management of chronic pain in this system.

For persistent (≥ 4 hours), moderate, and severe pain (NRS≥4), we analyzed possible related factors in detail. Injection, changing the dressing of the wound, and change of position/chest percussion

were the three most common care-related factors related to pain that were reported by patients. This finding is similar to the results of Coutaux et al.[36] and Ambrogi et al.,[21] which showed injections and change in position/chest percussion were the most common causes of care-related pain in inpatients. We found that pain caused by injections, change in position/chest percussion, and rehabilitation had all been reduced. Among these factors, the rate of injection pain decreased significantly over the three consecutive years (49.3%, 42.1%, 27.9%) of this study. Taken together, these results indicate that the pain nursing education and training that we regularly organized in wards and departments might have improved the pain caused by the aforementioned factors.

There are some limitations within the study. First, our findings lack generalizability because all study populations were drawn from a single hospital. Second, we did not ask patients about pain prior to admission, nor did we investigate the detailed causes of pain. Third, we relied only on recalling the patient's past pain through questionnaires, and there is no objective recording tool in the process. Fourth, for patients who were illiterate or had difficulty understanding, the interviewer would personally ask and assist in answering. Although the research team conducted rigorous and standardized training of the interviewers before conducting the survey, inevitably there could have been discrepancies between the patients' actual feelings and their answers. Our study results represent the overall pain prevalence of inpatients. Despite the above limitations, this study not only provides a detailed description of the characteristics of pain prevalence in inpatients, but also sheds light on the effectiveness of the pain management system.

Conclusions

Pain is a common complaint in all medical institutions. Our project effectively decreased the prevalence rate of pain among the hospitalized patients. The project included 4 core factors: (1) a

multidisciplinary team, (2) an evidence-based education program, (3) a real-time “3324” PDCA response cycle to control pain, and (4) a data-driven Information Dashboard to integrate teamwork. Herein, we propose a model of pain management “toward a pain-free hospital”. However, the results show the prevalence rate of pain up to 60% remains high. Hence, it is necessary to persevere in our efforts to improve the physical and mental condition of patients suffering from pain.

Acknowledgements

We thank Hui-Hsun Chang, Ren-Jyun Yan, Ren-Qi Chen, Yi-Ting Liao, Ying-Jyun Shih and Yun-Tong Sun as interviewers. They helped us interview inpatients and collect questionnaires

References

1. Melotti RM, Samolsky-Dekel BG, Ricchi E, Chiari P, Di Giacinto I, Carosi F, et al. Pain prevalence and predictors among inpatients in a major Italian teaching hospital. A baseline survey towards a pain free hospital. European Journal of Pain. 2005;9(5):485-95.
2. Subramanian P, Ramasamy S, Ng KH, Chinna K, Rosli R. Pain experience and satisfaction with postoperative pain control among surgical patients. International Journal of Nursing Practice. 2016;22(3):232-8.
3. Gregory J, McGowan L. An examination of the prevalence of acute pain for hospitalised adult patients: a systematic review. Journal of clinical nursing. 2016;25(5-6):583-98.
4. Pujatti PB. Prevalência de dor e adequação da terapia analgésica em um hospital público referência. Rev Med Minas Gerais. 2019;29(Supl 8):S65-S70.
5. Wu C-L, Hung Y-L, Wang Y-R, Huang H-M, Chang C-H, Wu C-C, et al. Pain prevalence in

hospitalized patients at a tertiary academic medical center: Exploring severe persistent pain. PloS one. 2020;15(12):e0243574.

6. Strohbuecker B, Mayer H, Evers GC, Sabatowski R. Pain prevalence in hospitalized patients in a German university teaching hospital. Journal of pain and symptom management. 2005;29(5):498-506.

7. Das SK, Dhar SS, Panigrahi A. Prevalence of pain and its characteristics in hospitalized patients in an Indian teaching hospital. Pain Management Nursing. 2020;21(3):299-303.

8. Visentin M. Towards a Pain-free Hospital: a project to improve the approach to the patient in pain. The Journal of Headache and Pain. 2002;3(2):59.

9. Maier C, Nestler N, Richter H, Hardinghaus W, Pogatzki-Zahn E, Zenz M, et al. The quality of pain management in German hospitals. Deutsches Ärzteblatt International. 2010;107(36):607.

10. Gordon DB, Dahl JL, Miaskowski C, McCarberg B, Todd KH, Paice JA, et al. American pain society recommendations for improving the quality of acute and cancer pain management: American Pain Society Quality of Care Task Force. Archives of internal medicine. 2005;165(14):1574-80.

11. Milewska M, Horosz B, Ladyko A. Pain free hospital: recommendation for the acute pain management in Poland. J Pain Relief. 2013;2(120):2167-0846.1000.

12. Ivers N, Jamtvedt G, Flottorp S, Young JM, Odgaard- Jensen J, French SD, et al. Audit and feedback: effects on professional practice and healthcare outcomes. Cochrane database of systematic reviews. 2012;(6).

13. Dowding D, Randell R, Gardner P, Fitzpatrick G, Dykes P, Favela J, et al. Dashboards for improving patient care: review of the literature. International journal of medical informatics. 2015;84(2):87-100.

14. Needham DM. Patient safety, quality of care, and knowledge translation in the intensive care unit. Respiratory care. 2010;55(7):922-8.

15. Reid C, Davies A. The World Health Organization three-step analgesic ladder comes of age.

Sage Publications Sage CA: Thousand Oaks, CA; 2004. p. 175-6.
16. Hanks G, Cherny NI, Christakis NA, Kaasa S. Oxford textbook of palliative medicine: Oxford university press; 2011.
17. Freye E. Opioids in medicine: a comprehensive review on the mode of action and the use of analgesics in different clinical pain states. 2008.
18. Gianni W, Madaio R, Di Cioccio L, D'Amico F, Policicchio D, Postacchini D, et al. Prevalence of pain in elderly hospitalized patients. Archives of gerontology and geriatrics. 2010;51(3):273-6.
19. Wadensten B, Fröjd C, Swenne CL, Gordh T, Gunningberg L. Why is pain still not being assessed adequately? Results of a pain prevalence study in a university hospital in Sweden. Journal of clinical nursing. 2011;20(5- 6):624-34.
20. Fabbian F, De Giorgi A, Pala M, Menegatti AM, Gallerani M, Manfredini R. Pain prevalence and management in an internal medicine setting in Italy. Pain Research and Treatment. 2014;2014.
21. Ambrogi V, Tezenas du Montcel S, Collin E, Coutaux A, Bourgeois P, Bourdillon F. Care-related pain in hospitalized patients: Severity and patient perception of management. European Journal of pain. 2015;19(3):313-21.
22. Xiao H, Liu H, Liu J, Zuo Y, Liu L, Zhu H, et al. Pain prevalence and pain management in a Chinese hospital. Medical science monitor: international medical journal of experimental and clinical research. 2018;24:7809.
23. Costantini M, Viterbori P, Flego G. Prevalence of pain in Italian hospitals: results of a regional cross-sectional survey. Journal of pain and symptom management. 2002;23(3):221-30.
24. Salomon L, Tcherny-Lessenot S, Collin E, Coutaux A, Levy-Soussan M, Legeron M-C, et al. Pain prevalence in a French teaching hospital. Journal of pain and symptom management. 2002;24(6):586-92.
25. Bovier PA, Charvet A, Cleopas A, Vogt N, Perneger TV. Self-reported management of pain

in hospitalized patients: link between process and outcome. The American journal of medicine. 2004;117(8):569-74.

26. Vallano A, Malouf J, Payrulet P, Baños JE, Hospital CRGfSPi. Prevalence of pain in adults admitted to Catalonian hospitals: a cross-sectional study. European Journal of pain. 2006;10(8):721-31.

27. Gerbershagen K, Gerbershagen HJ, Lutz J, Cooper-Mahkorn D, Wappler F, Limmroth V, et al. Pain prevalence and risk distribution among inpatients in a German teaching hospital. The Clinical journal of pain. 2009;25(5):431-7.

28. Fillingim RB, King CD, Ribeiro-Dasilva MC, Rahim-Williams B, Riley III JL. Sex, gender, and pain: a review of recent clinical and experimental findings. The journal of pain. 2009;10(5):447-85.

29. Gunnarsdottir S, Ward SE, Serlin RC. A population based study of the prevalence of pain in Iceland. Scandinavian Journal of Pain. 2010;1(3):151-7.

30. Tocher J, Rodgers S, Smith MA, Watt D, Dickson L. Pain management and satisfaction in postsurgical patients. Journal of clinical nursing. 2012;21(23-24):3361-71.

31. Bourdillon F, du Montcel ST, Collin E, Coutaux A, Lébeaupin E, Cesselin F, et al. Determinants of patient satisfaction regarding pain care. Revue d'epidemiologie et de sante publique. 2012;60(6):455-62.

32. Zoëga S, Ward SE, Sigurdsson GH, Aspelund T, Sveinsdottir H, Gunnarsdottir S. Quality pain management practices in a university hospital. Pain Management Nursing. 2015;16(3):198-210.

33. Rottenberg Y, Jacobs JM, Stessman J. Prevalence of pain with advancing age brief report. Journal of the American Medical Directors Association. 2015;16(3):264. e1-. e5.

34. Falk H, Henoch I, Ozanne A, Öhlen J, Ung EJ, Fridh I, et al. Differences in symptom distress based on gender and palliative care designation among hospitalized patients. Journal of Nursing Scholarship. 2016;48(6):569-76.

35. Stotts NA, Puntillo K, Morris AB, Stanik-Hutt J, Thompson CL, White C, et al. Wound care pain in hospitalized adult patients. Heart & lung. 2004;33(5):321-32.

36. Coutaux A, Salomon L, Rosenheim M, Baccard AS, Quiertant C, Papy E, et al. Care related pain in hospitalized patients: A cross- sectional study. European Journal of pain. 2008;12(1):3-8.

Supporting information

S1 Fig. The organizational structure of the Pain-Free Hospital Committee

S2 Fig. 3324 Dashboard system. We use the 3324 dashboard system to track patients' pain status and current prescriptions in real time, and pain experts could leave recommendations in the system after evaluation. This helped us improve the efficiency of pain management.

S1 Table. Conference for review of pain management system. Pain-free hospital group meetings are held regularly every two weeks to set up cross-team member participation, establish communication channels, and invite team members to discuss care implementation strategies. Monthly case discussions and ward team meetings were conducted for outpatient cases with special or refractory pain. The management committee discussed the business promotion, the work report of each group, the development of the annual policy, and the progress review on a quarterly basis, and reported the implementation progress to the hospital every six months.

S1 Appendix. Structured questionnaire for patient perception of pain care survey

S1 File. Questionnaire data from 2018 to 2020

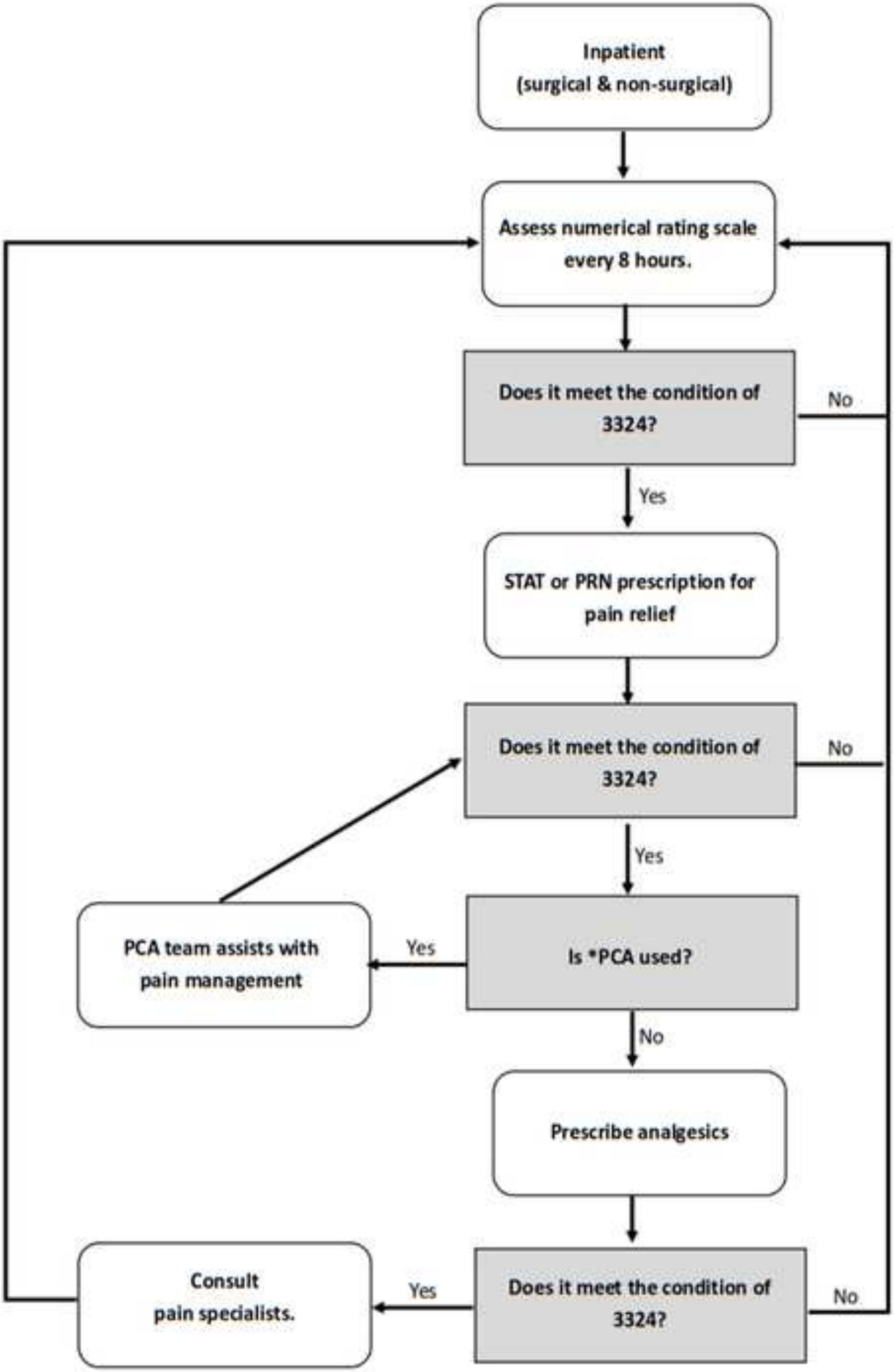
Inpatient
(surgical & non-surgical)
Assess numerical rating scale
every 8 hours.
Does it meet the condition of
3324?
No
Yes
STAT or PRN prescription for
pain relief
Does it meet the condition of
3324?
No
Yes
PCA team assists with
pain management
Yes
Is *PCA used?
No
Prescribe analgesics
Consult
pain specialists.
Yes
Does it meet the condition of
3324?
No

Fig 2

Click here to access/download;Figure;Fig2.tif.tif

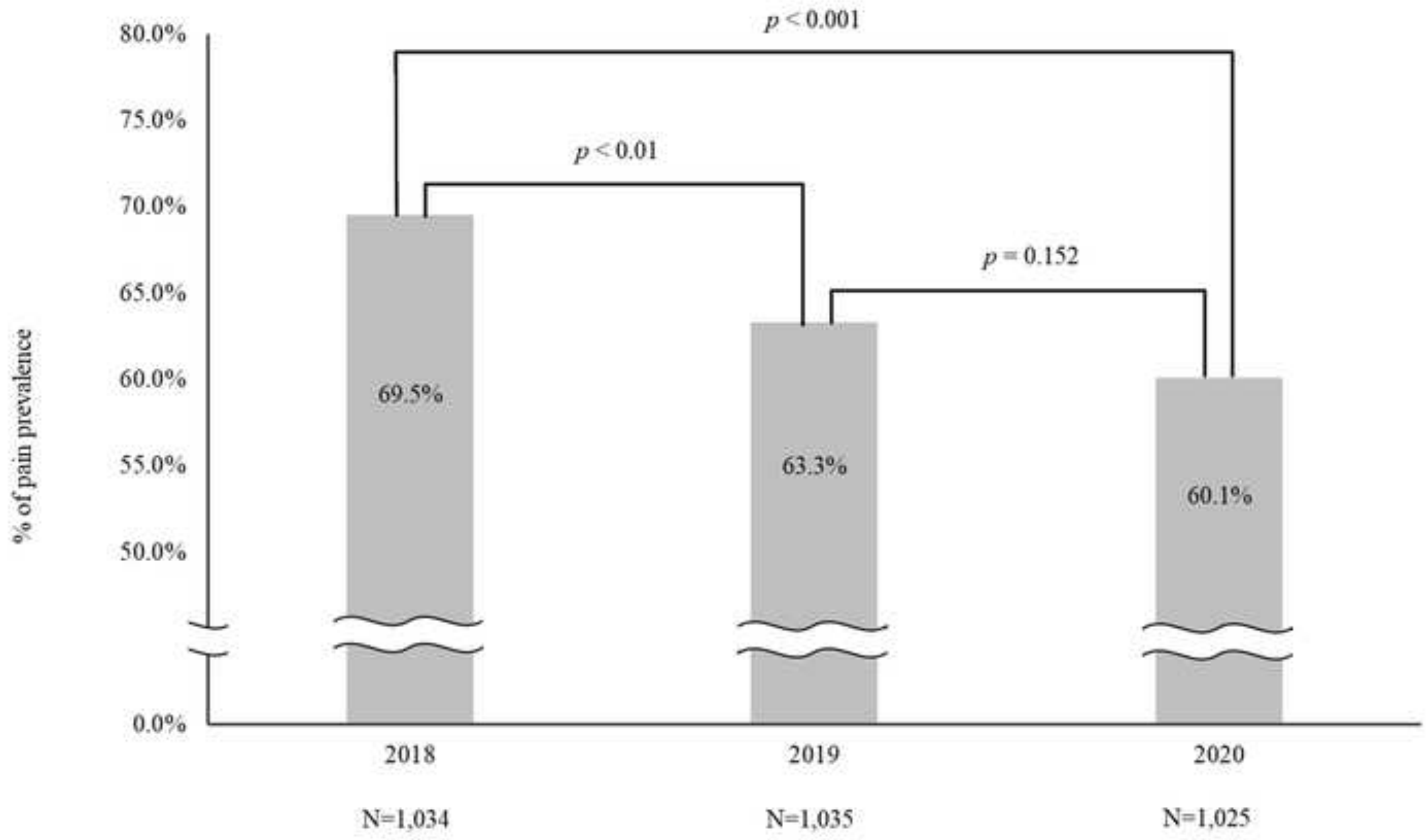

Fig 3

Click here to access/download;Figure;Fig3.tif.tif

Figure 3(a)

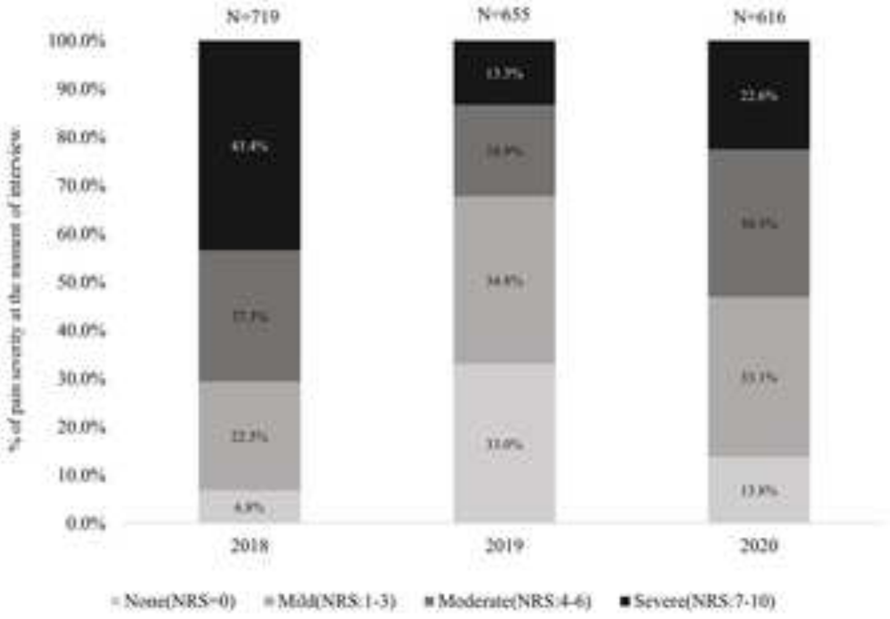

Figure 3(b)

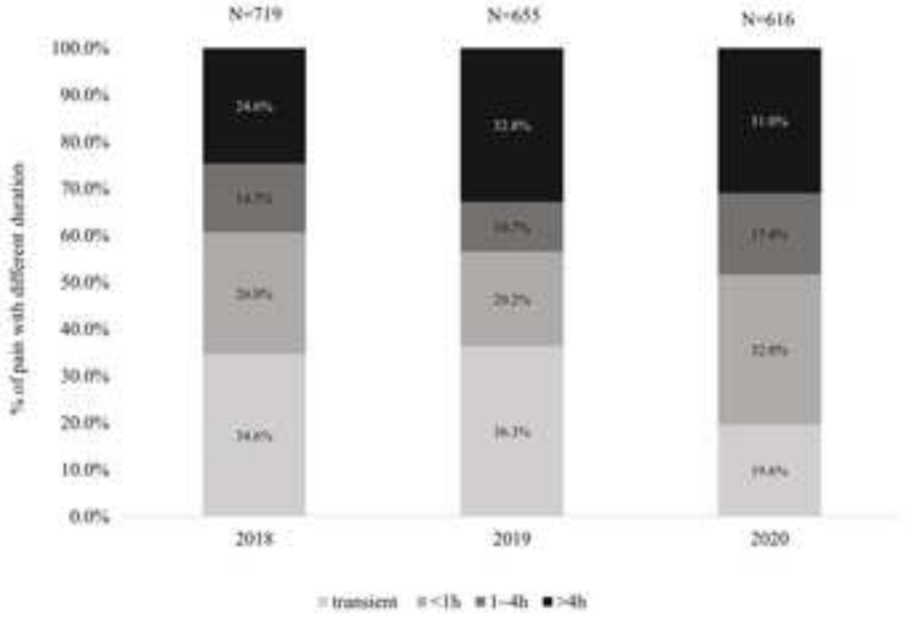

S1 Fig

Click here to access/download
Supporting Information
S1_fig.pdf.pdf

S2 Fig

Click here to access/download
Supporting Information
S2_fig.pdf.pdf

S1 Table

Click here to access/download
Supporting Information
S1_table.pdf.pdf

S1 Appendix

Click here to access/download

Supporting Information

S1_appendix.pdf.pdf

S1 File

Click here to access/download
Supporting Information
S1_file.xlsx.xlsx
