# Supplementary material for: Three-year hospital-wide pain management system implementation at a tertiary medical center: Pain prevalence analysis
Source: PLoS One. 2023 Apr 13;18(4):e0283520. doi: 10.1371/journal.pone.0283520 (PMC10101381; doi:10.1371/journal.pone.0283520)
Supplement: Attachment — Submitted filename: Response to Reviewers.docx [file pone.0283520.s007.docx]

Dear reviewer:

Thank you for inviting us to submit a revised draft of our manuscript entitled, “Three-year hospital-wide pain management system implementation at a tertiary medical center: pain prevalence analysis” to PLoS One. We also appreciate the time and effort you have dedicated to providing insightful feedback on ways to strengthen our paper. Thus, it is with great pleasure that we resubmit our article for further consideration. We have incorporated changes that reflect the detailed suggestions you have graciously provided. We also hope that our edits and the responses we provide below satisfactorily address all the issues and concerns you have noted.

To facilitate your review of our revisions, the following is a point-by-point response to the questions and comments delivered in your letter.

1. **Need more description of the intervention**

The intervention mainly included the development of standard procedures for inpatient pain management, the implementation of hospital-wide pain medicine education and training, the establishment of a dashboard system to track pain status, and regular audits and feedback. We have incorporated the above text into the manuscript.

2. **This looks like a typo. Please merge the sentences**

To gain an understanding of the changes in the prevalence of pain in inpatients under the care of the pain management system.

We have changed the above text to “This study aimed to gain an understanding of the changes in the prevalence of pain in inpatients under the care of the pain management system.”

3. **selected**

We randomly select eligible patients in the general ward.

To fit the proper tense, we have changed the above text to “We randomly selected eligible patients in the general ward.”

4. **See my comments above about the abstract. Please address these here.**

We have modified the content of the abstract according to your comments, thank you for your advice.

5. **What were the features of this campaign? Please describe these here.**

“Towards a Pain-Free Hospital” was first started in Montreal, Canada.* The features of the campaign are as following. (1) Pain is a problem common to all medical specialities. (2) Every hospital professional is faced with the problem of pain. (3) The whole hospital must be involved in the campaign. (4) The general public should be involved in the campaign as well.

*Besner G, Rapin CH (1993) The hospital. Creating a pain-free environment: a program to improve pain control in hospitalized patients, J Palliative Care 9:51–52

6. **How many beds in total?**

Taichung Veterans General Hospital (TCVGH) is a 1,500-bed teaching hospital and tertiary referral medical center in central Taiwan.

7. **Any that treat pain specifically?**

We don’t have dedicated wards for pain patients.

8. **This section needs more description. What are the specific aims of the project, what gap is it addressing? How did it inform the literature you reviewed?**

The purpose of this project is to comprehensively relieve pain and improve medical quality. We are committed to (1) using the same tool or technique for all medical staff to assess pain, at least 3 times a day, and actively treat pain, (2) documenting pain management in detail, (3) implementing pain medical education for all medical staff in the hospital and (4) monitoring the comorbidities associated with pain management.

We searched for literature on Towards a Pain-Free Hospital and hospital-wide pain management according to the hospital environment and conditions. We reviewed the recent literature to update our guidelines for pain management.

9. **Comprising how many members?**

There are 28 members in total, including the convener, deputy convener, 19 physicians, 3 nurses, 3 administrative staff and 1 information engineering staff. The organization chart for the Pain-Free Hospital Committee is as following.

The organizational structure of the Pain-Free Hospital Committee

10. **Is this the first time you use this acronym? if so, please write out in full.**

PDCA: plan-do-check-act

I've made the correction, thank you for the reminder.

11. **More description of 333 needed here. How is it relevant to what you have developed. Also it is for chronic pain, but you are trying to reduce all pain? Please clarify what was done for acute pain**

According to National Comprehensive Cancer Network (NCCN) guideline, to allow for dose titration, the short-acting opioids could be given as often as once per hour as needed, if pain is inadequately controlled. If hourly dosing is needed for more than 3 cycles, reassessment or other intervention is recommended. Therefore, we believe that the occurrence of breakthrough pain in patients should be controlled at less than 3 times a day. In addition, the patient's pain should be managed at a near-painless level (Visual Analogue Scale, VAS ≤ 3) with minimal side effects. Based on this, we had formulated the “3-3-3 principle” for chronic pain, which means that the patient's pain should be controlled to a VAS of 3 or less within 3 days of admission, and the frequency of breakthrough pain should be less than 3 times a day. In recent years, in response to the Towards a Pain-Free Hospital policy, we referred to the 3-3-3 principle and designed an approach termed “3324” for all pain in the hospital. This number means that the scale of VAS was > 3 and occurred ≥ 3 times within the last 24 hours during hospitalization. We wanted to treat acute and chronic pain with the same rigor. The data of patients who meet the criteria of 3324 will be displayed in the Dashboard Pain Management System, and the nurses and physicians can see it on the working panel of the electronic medical record system.

12. **Give some possible scenarios here. Example what happens if pain levels are high?**

For example, a patient suffers from postoperative wound pain. If the ward nurse assesses that the pain level exceeds 3 points, analgesics will be given according to the postoperative doctor's order of the surgeon, and guide the patient how to use the PCA pump. If the analgesic effect is poor, the PCA team of the pain department will be asked to adjust the dosage of the pump, or the ward physician will be asked to evaluate the situation and prescribe an analgesic drug. If the above treatments are still ineffective, or if the pain level is greater than three times within 24 hours, a pain specialist will be consulted to the ward to evaluate the pain and provide advice. Cases that meet 3324 will be automatically tracked in the dashboard pain management system through electronic records.

13. **What clinical care guidelines? You need to provide references here**

We have attached references in the article, thank you.

14. **Was the questionnaire administered at baseline? This needs to be clearly stated.**

Five experts evaluated this questionnaire with a Content Validity Index (CVI) of 0.90. Before the questionnaire survey, 30 inpatients were sampled in the general ward for pretest. Regarding the reliability, we used KR20 and Cronbach's α coefficient to test the questionnaire.

Since this study is not a scale-based questionnaire, only (1) I have side effects after taking painkillers (2) Patient satisfaction can be analyzed. I have side effects after taking painkillers with a KR20 pretest of 0.765 and our questionnaire of 0.704. The Cronbach’s α pretest value of care satisfaction was 0.838, and our questionnaire was 0.831.

15. **What were you comparing? be explicit about when you used these tests and for what**

We used the chi-square test to analyze the variability of the patient's demographic data and care-related pain with severity ≥ 4 and duration ≥ 4 hours. In addition, we used logistic regression to determine the factors related to pain and analyze trends in pain prevalence over the three years.

16. **This may have influenced results - particularly as you are not sampling the same patients over the 3-year period.**

Our sampling method tends to cause variability in the demographic data. We hope to reduce the bias, so we use logistic regression to analyze pain factors and pain prevalence trends. However, it may have influenced the results of the study. This is one of the limitations of our study and is written in the article. Thank you for your suggestion.

17. **Present the results and sample size for both years here**

We have presented the results and sample size. Figure 3(a), which we did not label in detail in the original manuscript, has been marked up. Thank you for your reminder.

18. **Add the statistical test that was used**

We used the chi-square test to analyze changes in the proportions of the various causes of care-related pain over a three-year period.

19. **How frequently were the training conducted? There needs to be more description on what you actually did early on in the methods as it is not clear. Other readers need to be able to replicate what you did so more detail is required**

For pain specialist training

(1) Case Discussion Meetings were held regularly every month.

(2) Pain physician instructed and demonstrated ultrasound-guided nerve block every week in the operation room.

(3) Trainees, resident doctors and interns, participate in weekly pain medicine classes. The content of the training includes pharmacology, differential diagnosis of pain, techniques of regional analgesia, etc.

For clinical staff training

We previously lacked standardized pain education and training. Since the Towards a Pain-free Hospital project, we began to develop painless education and training materials. In May 2018, the first edition of the pain teaching material was drawn up by Dr. Chih-Cheng Wu, the chief of the pain department. In June 2018, to train seed instructors, one from each department and two from each ward were assigned to attend the training. Afterward, these seed instructors returned to the department and ward for the first advocacy. In July 2018, pain physicians provided pain-free hospital guidance to all departments of the hospital, and the guidance was completed before the end of the year. Afterwards, our pain physicians hold regular lectures in various departments every year. Our guidance covers pharmacology, pain assessments, and dashboard pain management systems.

20. **In what way was it updated every year? how do you think this may have impacted the results as you are no longer delivering the same intervention**

We promoted Pain-Free Hospital in various units in our hospital every year. Our guidance for clinicians includes pharmacology, pain assessments and dashboard pain management systems. In addition to promoting the above content, we provided new information on analgesics, additional tools for evaluating pain, and updates of the dashboard system. In order to promote the quality of Pain-Free Hospital, we updated the training content of medicine and informatics. Although standard process for inpatient pain management have not changed, it is possible that these updated information could have affected the results. This is one of the limitations of this study and is written in the manuscript.

21. **This should be presented in the results**

The pain rate of patients in the department of surgery (n=920, N=1284, 71.7%) was significantly higher than that of patients in the department of internal medicine (n=803, N=1484, 54.8%), and patients who underwent surgery (n=1185, N=1580, 75.0%) had a higher rate of pain than patients who did not undergo surgery (n=805, N=1514, 53.2%)

We presented the results in Table 1, 2, which we did not label in detail in the original manuscript. Thank you for your reminder.

**In email, Review#1 Comments to the Author**

**Abstract:**

**Not to certain if this may be a typo, but a minor revision is required: The prevalence 28 rates of pain experienced by the patients in the 3-year period were 69.5% (2018) (reference)**

We changed it to “During the three-year period, the prevalence of pain was 69.5% (2018) (reference), 63.3% (2019) (OR:0.768, p<0.01), and 60.1% (2020) (OR:0.662, p <0.001).” Thank you for your advice.

**Methods:**

**-Exclusion criteria: could you provide some examples of critical illness (e.g. cancer, heart/renal failure, patients receiving chemotherapy) to help better understand those that were not suitable for the study. Was pregnancy also excluded for the trial?**

This is a misapplication of the wording and should be replaced by unstable medical condition, that refers to a state in which the condition is so unstable that pain cannot be clearly expressed, such as shock, coma, etc. Therefore, patients with pregnancy, cancer, and cardiovascular disease were not excluded unless they were unable to express themselves. Thanks for your helpful comments.

**-Monitoring of those administering/reviewing the pain management program: This does not require an amendment to the manuscript and/or study design, but be keen to hear comments if there would be scope to undertake check-ups on process (e.g. 3 monthly) together with the 1 year that was initially undertaken for the study.**

Some of our Pain-Free Hospital Management Committee comments on the program are as follows.

(1) Medical staff usually say to male patients that "pain is normal and must be tolerated". Medical staff need to minimize stereotypes. We should face up to everyone’s pain problem.

(2) Patients with advanced age and low education level are more difficult to use the numerical rating scale. It is recommended that medical staff choose appropriate assessment tools for different patients.

(3) Patients with severe pain and pain from injection and blood drawing are high-risk groups of side effects after taking analgesics. Medical staff should pay attention to the occurrence of side effects after taking analgesics.

(4) The interface of the information system should be tested and optimized as early as possible, so that the subsequent guidance and application will be smoother.

**Results:**

**Demographics-**

**Disease categories. Were any of the participants enrolled into the study present with multiple disease categories? it may be worthwhile to see if this could impact on the response rates for the survey**

**Ethnicity- Not listed in the demographics but be good to see the population being assessed**

This is a typo, and we changed it to “Medical department”, which refers to the department that provides primary care while hospitalized. This study did not investigate the patient's disease and race, and we hope to work on this part in the future.

**Results:**

**Was the prevalence rates (pain) 2020 (60.1%) with inclusion of numbers during covid? Did the researchers feel the management was affected during the covid period as the rates of moderate severe pain increased in 2020? This would be understandable given the covid pandemic and the challenges the health practitioners may have experienced to manage an influx of patients during this period.**

Because of COVID-19 outbreak, part of the ward was converted into a negative pressure isolation room. That part of the ward was excluded from sampling at the time, so 1,025 samples were collected in 2020 (N=1034 in 2018 and N=1035 in 2019). At that time, the workload of medical staff increased, so the work of pain management may be relatively neglected. We think this may be related to the elevated prevalence of moderate to severe pain in 2020.

Thank you for giving us the opportunity to strengthen our manuscript with your valuable comments and queries. We attach importance to your feedback and hope that these revisions persuade you to accept our submission.

Sincerely,

Dr. Ming-Chuan Chen

chen510156@gmail.com

Corresponding Author: Dr. Ching-Hui Shen

shench07@gmail.com
