# Supplementary material for: Three-year hospital-wide pain management system implementation at a tertiary medical center: Pain prevalence analysis
Source: PLoS One. 2023 Apr 13;18(4):e0283520. doi: 10.1371/journal.pone.0283520 (PMC10101381; doi:10.1371/journal.pone.0283520)
Supplement: Attachment — Submitted filename: Response to Reviewers.docx [file pone.0283520.s008.docx]

Dear reviewer:

Thank you for inviting us to submit a revised draft of our manuscript entitled, “Three-year hospital-wide pain management system implementation at a tertiary medical center: pain prevalence analysis” to PLoS One. We also appreciate the time and effort you have dedicated to providing insightful feedback on ways to strengthen our paper. Thus, it is with great pleasure that we resubmit our article for further consideration. We have incorporated changes that reflect the detailed suggestions you have graciously provided. We also hope that our edits and the responses we provide below satisfactorily address all the issues and concerns you have noted.

To facilitate your review of our revisions, the following is a point-by-point response to the questions and comments delivered in your letter.

1. It is important to describe the patient population more clearly.

The detailed description of patient population in the article is as follows.

This study is a three-year longitudinal survey. All inpatients in general wards of Taichung Veterans General Hospital were the research subjects. The patients were enrolled if they (1) were at least twenty years old, (2) had a hospital stay of at least three days, (3) had clear consciousness, and (4) provided consent to be included in this study. Patients were excluded if they were not able to express themselves e.g., had dementia, brain injury, or unstable medical condition, such as shock or unconsciousness.

In total, 1034, 1035, and 1025 patients were recruited by quota sampling from wards in 2018, 2019, and 2020, respectively. A total of 3094 inpatients completed the survey in this study, including 1679 males (54.3%) and 1415 females (45.7%). They were divided into six groups according to age, among which 789 patients (25.5%) were aged 60-69. They were divided into five groups according to body weight, among which 966 patients (31.2%) accounted for 60-69 kg. 898 patients (29.0%) had a college education or above, followed by senior high school (27.4%), elementary school (21.8%), junior high school (14.9%), and illiterate (6.9%). In terms of marital status, most of the 2380 (76.9%) were married, and the number of married people increased year by year, while the ratio of others (divorced, separated, widowed) decreased year by year. Patients from the Department of Internal Medicine (47.3%) and Department of Surgery (41.5%) were the main patients. Patients with (51.1%) and without (48.9%) undergoing surgery accounted for about half.

2. You refer to cancer guidelines, but were all the patients cancer patients? The management of cancer and non-cancer pain are quite different. Was the model you used applies to both groups of patients and if so, what is the justification for this?

In response to the Towards a Pain-Free Hospital policy, considering the resources of the hospital and the feasibility of the policy, we hoped to develop a set of methods to cooperate with the dashboard system to monitor and identify patients with difficulty in pain management. Thus, we referred to the 3-3-3 principle and designed an approach termed “3324” for all pain monitoring in the hospital. This number means that the scale of VAS was > 3 and occurred ≥ 3 times within the last 24 hours during hospitalization. The data of patients who meet the criteria of 3324 will be displayed in the Dashboard Pain Management System, and the nurses and physicians can see it on the working panel of the electronic medical record system. The medical staff then take the necessary actions to control the patients’ pain following the process. If the patient still has refractory pain, the case manager of pain (a nurse) can visit the patient and consult with a pain specialist (anesthesiology) to adjust the analgesic prescription or consider regional analgesic therapies, such as peripheral nerve block. We hope to use this to identify inpatients who have difficulty in pain management immediately, and consult pain physicians to provide individualized treatment based on the patient’s condition, so as to improve the quality of pain management in the hospital.

Thank you for giving us the opportunity to strengthen our manuscript with your valuable comments and queries. We attach importance to your feedback and hope that these revisions persuade you to accept our submission.

Sincerely,

Dr. Ming-Chuan Chen

chen510156@gmail.com

Corresponding Author: Dr. Ching-Hui Shen

shench07@gmail.com
